# Prevalence and determinants of anxiety and depression among children and adolescents undergoing cancer treatment at a tertiary hospital in a resource-limited setting: A cross-sectional study

**DOI:** 10.1097/MD.0000000000050413

**Published:** 2026-08-28

**Authors:** Misikir Tesfaye Dirre, Elias Tesfaye, Alemayehu Negash Torba, Bezaye Alemu Tsegaye, Yonas Tesfaye, Mubarek Abera Mengistie, Milki Tufa Feyisa, Puturos Puturos Reta, Tadios Teka Bireda, Tamirat Godebo Woyimo

**Affiliations:** a Department of Psychiatry, Jimma University, Jimma, Ethiopia; b Department of Pediatrics, Jimma University, Jimma, Ethiopia; c Department of Clinical Oncology, Jimma University, Jimma, Ethiopia; d Department of Clinical Oncology, Addis Ababa University, Addis Ababa, Ethiopia; e Department of Internal Medicine, Jimma University, Jimma, Ethiopia.

**Keywords:** anxiety, cancer, cancer treatment, children and adolescents, depression

## Abstract

Childhood cancer significantly impacts the mental health and overall well-being of children and adolescents. Mental health issues, such as depression and anxiety, are common among these children. These conditions can lead to long-term consequences, including poor psychosocial functioning, an increased risk of substance misuse, and chronic mental and physical health problems. This study assesses the prevalence of anxiety and depression, along with associated factors, among children and adolescents undergoing treatment for cancer at a tertiary center in Ethiopia in 2024. An institution-based cross-sectional study was conducted involving 206 consecutively selected children and adolescents. Data were collected from March 1 to May 13, 2024. Anxiety and depression were screened using the Revised Child Anxiety and Depression Scale. A consecutive sampling technique was employed. The Kobo Toolbox was used to collect data, which were then exported to the Statistical Package for the Social Sciences version 26.0 for analysis. Logistic regression analysis was performed to identify factors associated with anxiety and depression, with statistical significance declared at *P* < .05. Odds ratios and 95% confidence intervals (CI) were calculated to determine the presence and strength of associations. A total of 203 respondents participated in this study, resulting in a response rate of 98.6%. The overall prevalence of depression and anxiety was 39.4% (95% CI: 33–46%) and 45.3% (95% CI: 38–52%), respectively. Significant associations with depression were found for the following factors: age (13–18; adjusted odd ratio [AOR] = 2.62, 95% CI: 1.29–5.32), hospitalization duration >4 weeks (AOR = 3.31, 95% CI: 1.23–8.88), and illness duration exceeding 24 months (AOR = 3.22, 95% CI: 1.06–9.73). In addition, corticosteroid medication (AOR = 3.69, 95% CI: 1.21–11.22), comorbid illness (AOR = 2.81, 95% CI: 1.26–6.25), and severe pain (AOR = 3.69, 95% CI: 1.21–11.21) were significantly associated with anxiety. The prevalence of anxiety and depression among children and adolescents undergoing treatment for cancer is high. Therefore, early screening and intervention are necessary, particularly for those aged 13 to 18, those hospitalized for more than 4 weeks, those with illness durations exceeding 24 months, and those experiencing comorbid illnesses, severe pain, or receiving corticosteroid medication.

## 1. Introduction

Cancer is the uncontrolled growth of abnormal cells that invade healthy tissues and organs.^[1]^ Childhood cancers, which affect children and teenagers, include leukemias, brain and central nervous system tumors, lymphomas, and solid tumors such as neuroblastoma and Wilms tumor.^[2]^ These cancers often grow and spread rapidly, necessitating prompt and aggressive treatment.

Although childhood cancer is relatively rare compared to adult malignancies, it represents a significant global health issue. According to GLOBOCAN 2020, approximately 400,000 new cases of childhood cancer are diagnosed globally each year among individuals aged 0 to 19 years.^[3]^ The prevalence of new cases varies based on geographical location, socioeconomic status, and healthcare accessibility.^[4]^ In high-income countries, advancements in treatment have led to 5-year survival rates exceeding 80%, highlighting significant progress in cancer care. However, survival rates can differ markedly depending on the type of cancer, the stage at diagnosis, and resource availability. In low- and middle-income countries (LMICs), outcomes remain less favorable due to limited healthcare infrastructure and access to treatment.^[2–5]^

The psychological impact of cancer on young patients is profound, with depression and anxiety being 2 of the most common mental health concerns in this demographic.^[6]^ Anxiety disorders in children manifest as excessive worry, fear, or nervousness, significantly interfering with daily activities. In contrast, depression is characterized by persistent sadness, loss of interest in activities, and alterations in sleep, appetite, and energy levels.^[7]^ The long-term consequences of untreated anxiety and depression can lead to chronic mental and physical health issues, increased risk of substance misuse, and even early mortality.^[8]^

The presence of cancer alongside a mental health disorder can significantly impair a young patient’s ability to cope with the rigorous demands of cancer treatment, including chemotherapy, radiation, and surgery. A cancer diagnosis complicates these mental health challenges, particularly among vulnerable populations like children and adolescents.^[9]^ Depression has been identified as a major risk factor for shorter survival in cancer patients, leading to treatment rejection, decreased motivation, poor adherence to treatment protocols, and heightened perceptions of pain and side effects.^[3,5]^ Furthermore, these mental health challenges can hinder social development, academic performance, and engagement in age-appropriate activities, resulting in long-term developmental setbacks.^[10,11]^

Comprehensive care in pediatric oncology extends beyond the physical treatment of cancer to include the management of medical comorbidities and associated mental health issues.^[1]^ Integrating mental health support into cancer care is essential for improving overall treatment outcomes and quality of life for young patients. This holistic approach acknowledges that addressing psychological well-being is as crucial as managing the physical aspects of cancer treatment.

Several barriers impact mental health care for children with cancer in LMICs, including limited access to mental health services, cultural stigmatization of mental illness, and socioeconomic factors that hinder appropriate care. These challenges contribute to the underreporting of anxiety, depression, and other mental illnesses among affected children, resulting in inadequate support for those in need. Moreover, the emotional toll of cancer and its treatment can exacerbate feelings of isolation, hopelessness, and distress, negatively affecting quality of life and straining family dynamics.^[11,12]^

This research aims to examine the prevalence of depression, anxiety, and associated factors among children and adolescents undergoing treatment for cancer in resource-limited settings. By addressing these gaps, the study seeks to provide valuable insights into the mental health needs of this vulnerable population, informing comprehensive care strategies.

The findings of this study may have significant implications for policy and practice, guiding the development of interventions to support mental health among children and adolescents undergoing cancer treatment in LMICs. By improving our understanding of the psychological challenges faced by these young patients, healthcare providers can better tailor their approaches to enhance overall patient care and outcomes.

## 2. Methods

### 2.1. Study design and setting

This study was conducted at Jimma Medical Center, located in Jimma town, Oromia region, approximately 352 km southwest of Addis Ababa, Ethiopia. As one of the oldest public hospitals in the country, Jimma Medical Center serves as the sole teaching and referral hospital for the southwestern region, catering to a diverse patient population. Annually, the hospital manages around 15,000 inpatient admissions, 160,000 outpatient visits, 11,000 emergency cases, and 4500 deliveries, drawing from a catchment area of about 1.5 million people. The center offers a wide range of medical services, with a particular focus on specialized care.

Among its various departments, the Pediatric Hematology/Oncology ward plays a crucial role in providing comprehensive services to pediatric cancer patients. Established in May 2016 through a collaboration between Jimma University and the USA-based Aslan Project, this unit is equipped with 28 beds and a dedicated team that includes oncologist, senior pediatricians specializing in oncology, residents, and nursing staff.

The study utilized an institutional-based cross-sectional design to examine the experiences and outcomes of children and adolescents aged 8 to 18 years who were currently receiving cancer treatment at the facility. Data collection occurred over a defined study period from March 1, 2024, to May 13, 2024, focusing on a consecutively selected population of pediatric patients attending the oncology clinic. This approach ensured that the findings accurately reflect the realities and challenges faced by young cancer patients in this region.

### 2.2. Eligibility criteria

All children aged 8 to 18 years with histologically confirmed malignancy and receiving anticancer treatment during the designated study period at the pediatric oncology clinic, Jimma University Medical Center (JUMC), were included in the research. This encompassed a diverse range of cancer types and treatment regimens, allowing for a comprehensive understanding of the pediatric oncology population. However, children who were in critically ill condition and unable to communicate effectively were excluded from the study. This exclusion was critical to ensure the reliability of the data collected and the safety of participants, as their inability to engage in communication would hinder informed consent and the ability to participate effectively in the study.

### 2.3. Sample size and sampling procedures

In this study, a thorough sampling technique was employed to ensure a representative sample of the pediatric oncology population at JUMC. The sample size was calculated using the formula:


$$\text{n}=Z^{\text{2}}p\;\;\;(1-p)/d^{\text{2}}$$

where a proportion of 50% was selected due to the lack of comparable studies in the region. With a confidence level of 95% and a margin of error of 5%, the standard normal variable Z was determined to be 1.96. Plugging these values into the formula yielded an initial sample size of 384. However, since the source population of eligible patients was fewer than 10,000, a correction formula was applied to refine the sample size to 193 after accounting for the estimated population during the study period. To ensure the study’s robustness, a 10% nonresponse rate was factored in, resulting in a final sample size of 206, which was then proportionally distributed between inpatient and outpatient pediatric oncology clinic participants.

To achieve this sample, a consecutive sampling technique was utilized, which involved the systematic enrollment of eligible children as they presented for treatment at the clinic.

### 2.4. Data collection tools and procedure

#### 2.4.1. Data collection tools

Anxiety and depression in children and adolescents was measured by the Revised Child Anxiety and Depression Scale (RCADS). The RCADS-25 is a validated measure that yields DSM-aligned subscale scores for anxiety and depression.^[13]^ Respondents rate the frequency of symptoms on a 4-point scale (0 = “Never” to 3 = “Always”). The raw scores for the anxiety and depression subscales were calculated separately. As per the standardized RCADS scoring manual, these raw scores were then converted to T-scores, which are gender-normed and age-normed scores that allow for comparison with a normative population. For the purpose of this study and to determine the prevalence of clinically significant symptoms, we operationalized “caseness” using the established clinical cutoff points. Participants with a T-score >70 on the depression subscale were classified as having depression, and those with a T-score >70 on the anxiety subscale were classified as having anxiety.^[13,14]^ This T-score > 70 threshold indicates a high severity of symptoms that lies above the clinical threshold.

The original English version has demonstrated good reliability with Cronbach α ranging from 0.71 to 0.88, and good validity by means of good model fit for the factor structure and high correlations with other depression and anxiety scales.^[13]^ RCADS exhibited robust internal consistency reliability in diverse assessment settings, nations, and languages. Specifically, the RCADS demonstrated strong internal consistency reliability across various evaluation contexts, nations, and dialects. Moreover, the meta-analysis of alpha coefficients yielded exceptional reliability, as evidenced by a mean alpha of .93 for the RCADS full scale.^[13,14]^ The internal consistency, Cronbach alpha coefficient of RCADS in the current study was 0.92. In our study the score was translated to local languages (both Amharic and oromifa) and also reliability was checked locally.

The Eastern Cooperative Oncology Group (ECOG) Scale was used to measure the patient’s performance status. This scale captures patient-derived functional status data on a scale of 0 to 4. An ECOG score of 2 to 4 indicates a poor performance status, whereas a score of 0 to 1 indicates a good performance status. Researchers have confirmed the validity of the ECOG Chinese version in assessing the performance status of Chinese patients with cancer.^[15]^

In the context of cancer pain, it is estimated that approximately 66% of cancer patients experience pain at some point during their illness.^[16]^ The area under the receiver operator characteristic curve for the numerical rating scale as a test for pain that interferes with functioning was 0.76, indicating fair accuracy. A pain screening numeric rating scale score of 1 was 69% sensitive (95% confidence intervals [CI] 60–78) for pain that interferes with functioning. Multilevel likelihood ratios for scores of 0, 1 to 3, 4 to 6, and 7 to 10 were 0.39 (0.29–0.53), 0.99 (0.38–2.60), 2.67 (1.56–4.57), and 5.60 (3.06–10.26), respectively. Results were similar when numeric rating scale scores were evaluated against the alternate definition of clinically important pain (pain that motivates a physician visit).^[17]^

Intensity of cancer pain was assessed using universal pain screening numerical rating scale with a 0 to 10 pain intensity on the Numerical Rating Scale. Pain intensity was categorized as “none” for a score of 0, “mild” for a score of 1 to 3, “moderate” for a score of 4 to 6, and “severe” for a score of 7 to 10 as reported by the patient.^[18,19]^

#### 2.4.2. Data collection procedure

A checklist was developed from reviews of several standard pieces of literature and written in English. The data was pretested and adjusted accordingly. A comprehensive 2-day training was provided to the data collectors by the principal investigator. The training covered the objectives of the study, the detailed administration protocol for each tool (including the RCADS, ECOG, and pain scale), techniques for interviewing children and adolescents, ethical conduct (including obtaining assent/consent and ensuring confidentiality), and methods to handle distressed participants. To ensure inter-rater consistency, the data collectors practiced administering the tools through role-playing and discussed scoring to reach a consensus on any ambiguous items. The data was collected through an interviewer-administered structured questionnaire via the Kobo Toolbox. Two trained psychiatry nurses collected data over two and a half months, with regular supervision and cross-checking by the supervisor and principal investigator to maintain uniformity. The use of interviewer-administered questionnaires ensured that children with varying literacy levels were able to participate fully and that the questions were understood uniformly.

### 2.5. Data analysis and data quality control procedure

The collected data was exported to Statistical Package for Social Science V.26.0 for analysis. Frequencies, percentages and summary statistics were calculated to define the study population relevant variables. Logistic regression analysis was employed to determine association between the outcome and predictor variables. Bivariate analysis was performed, and all variables with a *P*-value <.25 were selected as candidates for the multivariable logistic regression analysis. This relaxed significance level is recommended for initial screening to avoid prematurely excluding variables that may become significant in the adjusted model.^[20]^ Model fitness was checked by Hosmer and Lemeshow test and the *P*-value was 0.18, showing that the data set was best fit for the model. Multicollinearity was checked, variance inflation factor was <1.09 and a tolerance was >0.91. Then multivariable logistic regression was employed to identify predictors of dependent variable at *P* value < .05. Odds ratios and 95% CI was used to identify the presence and strength of association.

To assure the data quality high emphasis was given in designing data collection instrument. Questionnaire prepared in English was translated to local Language Afaan Oromo and Amharic by skilled language professionals and was back translated to English by independent skilled mental health professionals who are blind for English version to check its consistency. Two-day training for data collectors was given about data collection procedure and how to handle ethical issues. All data collectors had masters in clinical psychiatry and were practicing in psychiatry department of JUMC. Pretest was conducted on 5% (10) of the sample size 1 week before the main study at JUMC the pretest collected was not included in the analysis as part of the main study. Based on the pretest findings, questionnaires were updated. Regular supervision by the supervisors (senior psychiatrists, oncologists, and public health professionals) and principal investigator was made to ensure that all necessary data were properly collected. The collected data was reviewed and checked for completeness before data entry.

### 2.6. Ethical consideration

Ethical clearance was obtained from the Ethical Review Board of Institute of Health Sciences, Jimma University number of JUIH/IRB/056/24. Written informed consent was obtained from the parents or legal guardians of all participants. In addition, written assent was obtained from children and adolescents who were developmentally capable of providing assent after receiving age-appropriate information about the study. All right was given to the study participant’s caregiver/guardian to refuse or discontinue participation at any time they want and the chance to ask anything about the study. Confidentiality was maintained throughout the study period. The privacy and anonymity of study subjects was preserved by omitting their names from the questionnaire and storing their data in a secure location. This study was conducted in accordance with the guidelines of Good Clinical Practice and the Principles of the Declaration of Helsinki.

## 3. Results

### 3.1. Socio-demographic factors

A total of 203 participants were included during the final analysis, with a response rate of 98.6%. Among the participant, 114 (56.2%) of them were in the age group between 8 and 12 years with mean age of 11.25 (SD = 3.03) years, 134 (66%) were male and 145 (71.4%) were primary school students. Majority (73.4%) reside in rural area. 127 (62.6%) of them were middle child in birth order and about 91.1% reports they have a good relationship with their caregiver (Table 1).

**Table 1:** Socio-demographic characteristics of study participants undergoing treatment for cancer at Jimma medical center, Ethiopia 2024 (n = 203).

Variable	Category	Frequency (n)	Percentage (%)
Age	8–12 yrs	114	56.2
	13–18 yrs	89	43.8
Sex	Male	134	66
	Female	69	34
Educational status	Never joined	15	7.4
	Kindergarten	25	12.3
	Primary school	145	71.4
	secondary school	18	8.9
Residency	Jimma town	54	26.6
	Outside Jimma town	149	73.4
Birth order	First child	60	29.6
	Middle child	127	62.6
	Last child	16	7.9
Relationship with the caregiver or guardians	Good	185	91.1
	Intermediate	11	5.4
	Poor	7	3.4
discontinued education because of the illness	Yes	190	93.6
	No	13	6.4

### 3.2. Caregiver related factors

Among the participants, 86 (42.4%) of the caregiver were found in the age between 36 and 45 years. The mean age of caregiver was 43.1 years with an SD of ± 11.06. Majority (74.9%) of them were married, and 62.6% of them were female. Regarding educational status and occupation, about 43.3% of them had no formal education and 44.3% of the caregiver was farmer. 24 (11.8%) of the participant had family history of mental illness. 103 (50.7%) of the participant income were below poverty line (with an average monthly income <3707.4 ETB) based on World bank poverty line cut point < 2.15$/day (Table 2).

**Table 2:** Caregiver related factors of study participants undergoing treatment for cancer at Jimma medical center, Ethiopia 2024 (n = 203).

Variable	Category	Frequency (n)	Percentage (%)
Age of the caregiver	15–25 yrs	16	7.9
	26–35 yrs	29	14.3
	36–45 yrs	86	42.4
	>45 yrs	72	35.5
Sex of the caregiver	Female	127	62.6
	Male	76	37.4
Education level of the caregiver	Not formal education	88	43.3
	Primary (1–8)	32	15.8
	Secondary (9–10)	45	22.2
	Technical and vocational	17	8.4
	College and above	21	10.3
Marital status of the caregiver	Married	152	74.9
	Single	30	14.8
	Divorced	21	10.3
Occupation of the caregiver	Governmental employee	36	17.7
	Daily laborer	14	6.9
	Merchant	32	15.8
	Farmer	90	44.3
	Other	12	5.9
Family size	<3	40	19.7
	4–6	81	39.9
	>7	82	40.4
Family history of mental illness	Yes	24	11.8
	No	179	88.2
Income*	Below poverty line	103	50.7
	Above poverty line	100	49.3

Other occupations: Private work, health professional, driver, and teacher.

* Based on the World Bank poverty line cut point, the current US dollar to birr exchange rate of 1$ = 57.48.

### 3.3. Clinical related factors

Among study participant, 75 (36.9%) were first diagnosis cancer at age of < 8 years old. Regarding type of cancer, 63 (31%) of them had acute lymphoblastic leukemia and 39 (19.2%) of them had Hodgkin lymphoma. Majority (71.4%) of the study participant were on chemotherapy and 174 (85.7%) of them were taking corticosteroid medication. Almost all (95.6%) study participant had history of hospitalization and half (53.2%) of study participant were hospitalized for more than 4 weeks. Out of study participant, 88 (43.3%) of them had comorbid illness. Majority (56.7%) of study participant had poor performance status (Table 3).

**Table 3:** Clinical related factors of study participants undergoing treatment for cancer at Jimma medical center, Ethiopia 2024 (n = 203).

Variables	Category	Frequency (n)	Percentage (%)
Age at first diagnosis	1.<8 yrs old	75	36.9
	2.8–12	64	31.5
	3.13–18	64	31.5
Type of cancer	1.Acute lymphoblastic leukemia	63	31
	2.Osteosarcoma	20	9.9
	3.Hodgkin lymphoma	39	19.2
	4.Non Hodgkin lymphoma	29	14.3
	5.Nasopharyngeal carcinoma	13	6.4
	6.Wilms tumor	17	8.4
	7.Ewing sarcoma	12	5.9
	8.Others	10	4.9
Duration of the illness	1.<6 mo	80	39.4
	2.7–12 mo	49	24.1
	3.13–24 mo	43	21.2
	4.>24 mo	31	15.3
Type of treatment	1.Chemotherapy with Radiotherapy	14	6.9
	2.Chemotherapy	145	71.4
	3.Combination therapy	44	21.7
Corticosteroid medication	1.Yes	174	85.7
	2.No	29	14.3
Medication discontinuation for last 1 mo	1.Yes	18	8.9
	2.No	185	91.1
History of hospitalization	1.Yes	194	95.6
	2.No	9	4.4
Duration of hospitalization	1.<2 wks	33	16.3
	2.2–4 wks	53	26.1
	3.>4 wks	108	53.2
Comorbid illness	1.Yes	88	43.3
	2.No	115	56.7
Type of the comorbid illness	1.Acute malnutrition	39	19.2
	2.Hospital acquired infection	15	7.4
	3.Community acquired infection	11	5.4
	4.Acute gastroenteritis	13	6.4
	5.Other	10	4.9
Pain score	1.No pain	94	46.3
	2.Mild pain	57	28.1
	3.Moderate pain	23	11.3
	4.Severe pain	29	14.3
ECOG Performance Status	1.Good performance	88	43.3
	2.Poor performance	115	56.7

Other type of cancer: neuroblastoma, retinoblastoma, rhabdomyosarcoma, and acute myeloid leukemia. Other type of the comorbid illness: quadriparesis, measles, and tinea capitis.

ECOG = Eastern Cooperative Oncology Group.

### 3.4. Prevalence of depression among children and adolescent undergoing treatment for cancer

**Figure 1.**
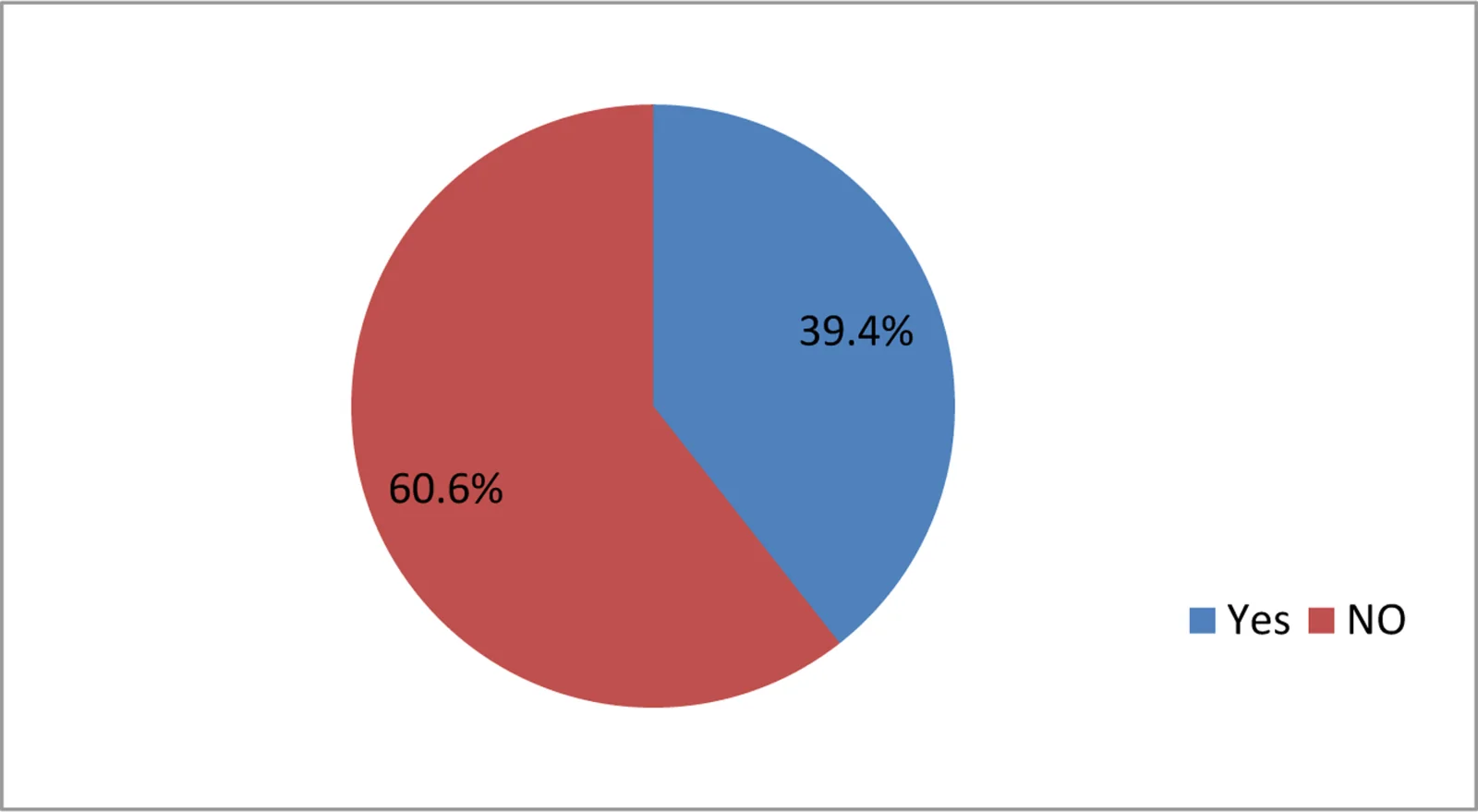
Prevalence of depression among children and adolescent undergoing treatment for cancer at Jimma medical center, Ethiopia 2024.

The overall prevalence of depression among study participants was 39.4% (95% CI: 33–46%; Fig. 1).

### 3.5. Prevalence of anxiety among children and adolescent undergoing treatment for cancer

**Figure 2.**
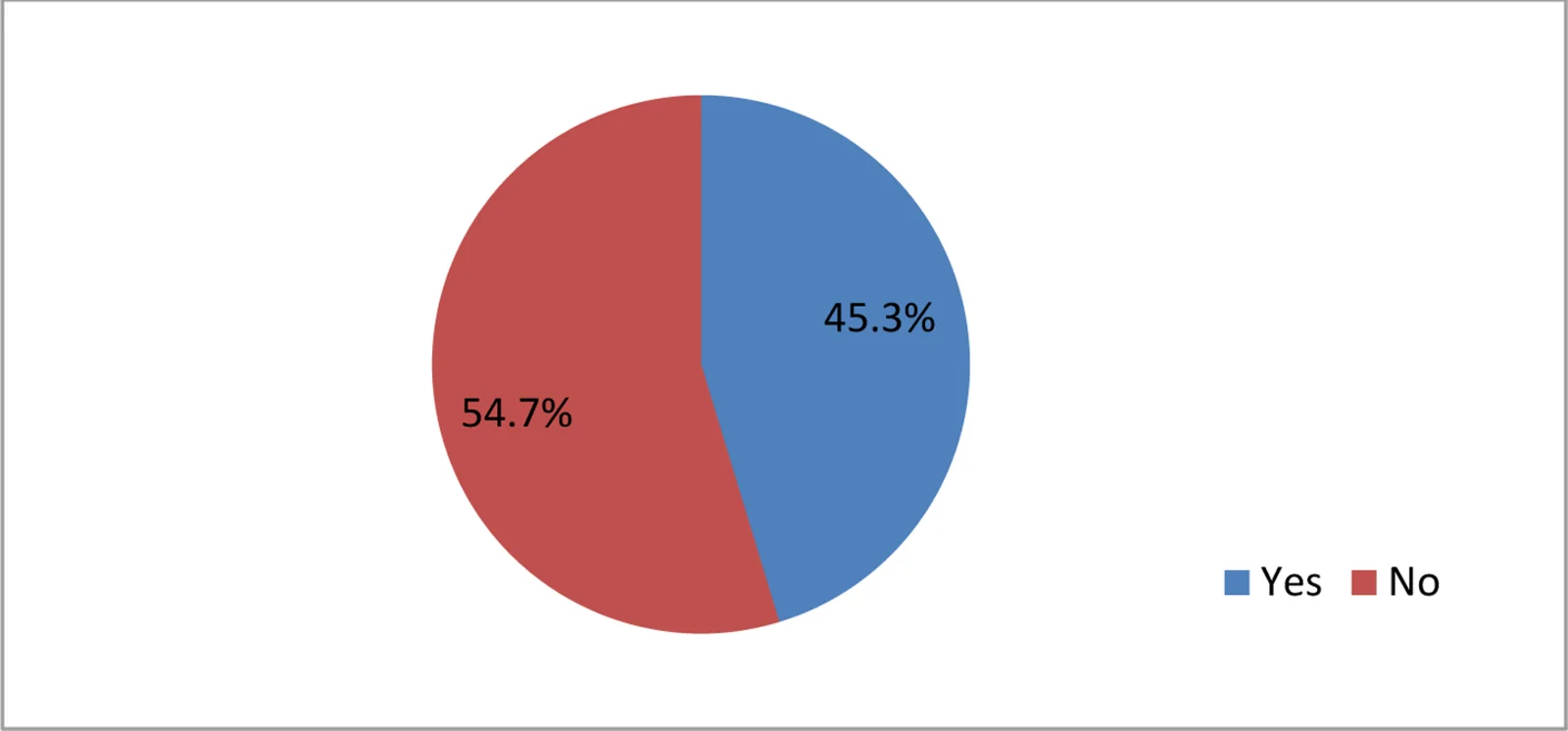
Prevalence of anxiety among children and adolescent undergoing treatment for cancer at Jimma medical center, Ethiopia 2024.

The overall prevalence of anxiety among study participants was 45.3% (95% CI: 38–52%; Fig. 2).

### 3.6. Factors associated with depression

#### 3.6.1. Bivariable logistic regression analysis

Upon bivariable logistic regression analysis age of children and adolescent, type of cancer, duration of hospital stays, educational status of the child and adolescent, duration of the illness, and discontinuation of school were found to be associated with depression and entered into multivariate analysis. Multivariable logistic regression analyses have revealed that age (13–18), >4 week of hospitalization and >24 months duration of illness was significantly associated with depression (Table 4).

**Table 4:** Multivariable logistic regression analysis of factors associated with depression among children and adolescent undergoing treatment for cancer at Jimma medical center, Ethiopia 2024 (N = 203).

Variables	Category	Depression		COR (95% CI)	AOR (95% CI)	*P*-value
		Yes	No			
Age of children and adolescent	8–12 yrs	33 (41.3)	81 (65.9)	1	1	
	13–18 yrs	47 (58.8)	42 (34.1)		2.62 (1.29–5.32)	.008*
Type of cancer	Acute lymphoblastic leukemia	24 (30)	39 (31.7)	1.43 (0.33–6.09)	0.72 (0.14–3.69)	.694
	osteosarcoma	6 (7.5)	14 (11.4)	1 (0.19–5.24)	0.576 (0.08–3.83)	.569
	Hodgkin lymphoma	16 (20)	23 (18.7)	1.62 (0.36–7.24)	1.21 (0.21–6.96)	.827
	Non Hodgkin lymphoma	20 (25)	9 (7.3)	5.18 (1.08–24.79)	2.758 (0.428–17.763)	.286
	Nasopharyngeal carcinoma	3 (3.8)	10 (8.1)	0.7 (0.11–4.53)	0.53 (0.06–4.49)	.56
	Wilms tumor	7 (8.8)	10 (8.1)	1.63 (0.31–8.61)	1.13 (0.16–7.97)	.89
	Ewing sarcoma	1 (1.3)	11 (8.9)	0.21 (0.01–2.46)	0.18 (0.01–2.48)	.2
	Others	3 (3.8)	7 (5.7)	1	1	
Educational status of the child and adolescent	Never joined	8 (10)	7 (5.7)	1	1	
	Kindergarten	14 (17.5)	11 (8.9)	1.11 (0.31–4.02)	1.126 (0.2–6.13)	.891
	Primary school	52 (65)	93 (75.6)	0.48 (0.16–1.42)	0.44 (0.07–2.05)	.3
	Secondary school	6 (7.5)	12 (9.8)	0.43 (0.11–1.79)	0.18 (0.02–1.27)	.087
Duration of the illness	<6 mo	29 (36.3)	51 (41.5)	1	1	
	7–12 mo	19 (23.8)	30 (24.4)	1.11 (0.53–2.31)	1.1 (0.42–2.85)	.83
	13–24 mo	13 (16.3)	30 (24.4)	0.762 (0.34–1.68)	1.02 (0.396–2.62)	.96
	>24 mo	19 (23.8)	12 (9.8)	2.78 (1.18–6.54)	3.22 (1.06–9.73)	.03*
Duration of hospital stay	< 2 wks	8 (10)	25 (20.3)	1	1	
	2–4 wks	17 (21.3)	36 (29.3)	1.47 (0.55–3.94)	2.19 (0.65–7.29)	.2
	>4 wks	51 (63.7)	57 (46.3)	2.79 (1.15–6.74)	3.31 (1.23–8.88)	.02*
Discontinued education because of the illness	Yes	78 (97.5)	112 (91.1)	3.83 (0.82–17.76)	4.39 (0.58–33.13)	.15
	No	2 (2.5)	11 (8.9)	1	1	

1 = reference categories, AOR: adjusted odd ratio, CI = Confidence interval, COR = crude odd ratio.

* Significance at 5% level.

The odds of developing depression were 2.62 times higher among age group between 13 and 18 years compared with those whose age category is 8 and 12 years old (adjusted odd ratio [AOR] = 2.62, 95% CI: 1.29–5.32). Likewise, children and adolescent who had > 4 weeks of hospitalization were 3.31 times more likely to develop depression than those who had < 2 weeks of hospitalization (AOR = 3.31, CI: 1.23–8.88).

In addition, the odds of developing depression were 3.22 times higher among study participant who had > 24 months of duration of illness than whose had < 6-month duration of illness (AOR = 3.22, 95% CI: 1.06–9.73).

### 3.7. Factors associated with anxiety

#### 3.7.1. Bivariable logistic regression analysis

Upon bivariable logistic regression analysis age of children and adolescent, type of cancer, educational status of the child and adolescent, educational status of caregiver, corticosteroid medication, comorbid illness, sex of caregiver, and severity of pain and discontinuation of school were found to be associated with anxiety and entered into multivariable analysis. Multivariable logistic regression analyses have revealed who had comorbid illness; severe pain and corticosteroid medication were significantly associated with anxiety (Table 5).

**Table 5:** Multivariable logistic regression analysis of factors associated with anxiety among children and adolescent undergoing treatment for cancer at Jimma medical center, Ethiopia 2024 (N = 203).

Variables	Category	Anxiety		COR (95% CI)	AOR (95% CI)	*P*-value
		Yes	No			
Age of children and adolescent	8–12 yrs	56 (60.9)	58 (52.3)	1	1	
	13–18 yrs	36 (39.1)	53 (47.7)	0.7 (0.4–1.23)	0.48 (0.23–1.03)	.06
Type of cancer	Acute lymphoblastic leukemia	34 (37)	29 (26.1)	1.17 (0.31–4.45)	2.06 (0.36–11.83)	.41
	osteosarcoma	7 (7.6)	13 (11.7)	0.583 (0.11–2.51)	1.31 (0.18–9.13)	.78
	Hodgkin lymphoma	16 (17.6)	23 (20.7)	0.69 (0.17–2.81)	1.56 (0.24–10.03)	.63
	Non Hodgkin lymphoma	7 (7.6)	22 (19.83)	0.31 (0.07–1.43)	0.39 (0.06–2.41)	.69
	Nasopharyngeal carcinoma	10 (10.9)	3 (2.7)	3.33 (0.55–19.94)	5.42 (0.56–52.1)	.14
	Wilms tumor	8 (8.7)	9 (8.)	0.88 (0.18–4.24)	1.69 (0.21–13.24)	.61
	Ewing sarcoma	5 (5.4)	7 (6.3)	0.71 (0.13–3.86)	0.99 (0.11–8.99)	.99
	Others	5 (5.4)	5 (4.5)	1	1	
Educational status of the child and adolescent	Never joined	7 (7.6	8 (7.2)	1	1	
	Kindergarten	15 (16.3)	10 (9)	1.71 (0.47–6.24)	2.01 (0.43–9.39)	.37
	Primary school	59 (64.1)	86 (77.5)	0.78 (0.27–2.27)	0.65 (0.16–2.66)	.55
	secondary school	11 (12)	7 (6.3)	1.79 (0.44–7.19)	4.29 (0.71–25.91)	.11
Educational status of caregiver	Not formal education	39 (42.4)	49 (44.1)	0.72 (0.27–1.87)	1.23 (0.38–4.01)	.72
	Primary school	21 (22.8)	11 (9.9)	1.73 (0.56–5.34)	3.19 (0.78–13.01)	.11
	Secondary school	14 (15.2)	31 (27.9)	0.41 (0.14–1.19)	0.55 (0.14–2.04)	.37
	Technical and vocational	7 (7.6)	10 (9)	0.63 (0.17–2.31)	1.00 (0.18–5.4)	.99
	College and above	11 (12)	10 (9)	1	1	
Corticosteroid medication	Yes	85 (92.4)	89 (80.2)	3 (1.21–7.39)	3.69 (1.21–11.22)	.02*
	No	7 (7.6)	22 (19.8)	1	1	
discontinued education because of the illness	Yes	84 (91.3)	106 (95.5)	0.49 (0.15–1.57)	1.01 (0.22–4.56)	.99
	No	8 (8.7)	5 (4.5)	1	1	
Comorbidity illness	Yes	54 (58.7)	34 (30.6)	3.21 (1.8–5.74)	2.81 (1.26–6.25)	.01*
	No	38 (41.3)	77 (69.4)	1	1	
Sex of caregiver	Male	63 (68.5)	64 (57.7)	1	1	
	Female	29 (31.5)	47 (42.3)	0.62 (0.35–1.11)	0.60 (0.28–1.25)	.17
Severity of Pain	No pain	37 (40.2)	57 (51.4)	1	1	
	Mild pain	26 (28.3)	31 (27.9)	1.29 (0.66–2.51)	0.91 (0.36–2.32)	.85
	Moderate pain	11 (12)	12 (10.8)	1.41 (0.565–3.53)	1.58 (0.51–4.93)	.42
	Severe pain	18 (19.6)	11 (9.9)	2.25 (1.07–5.93)	3.69 (1.21–11.21)	.02*

1 = reference categories, AOR = adjusted odd ratio, CI = confidence interval, COR = crude odd ratio.

* Significance at 5% level.

The odds of developing anxiety were 3.69 times higher among who used corticosteroid medication compared with those whose had not use (AOR = 3.69, 95% CI: 1.21–11.22). In addition, the odds of developing anxiety were 2.81 times higher among who had comorbid illness than those who had not (AOR = 2.81, 95% CI: 1.26–6.25).

Furthermore, The odds of developing anxiety were 3.69 times higher among children and adolescent who had severe pain compared to those who had no pain (AOR = 3.69, 95% CI: 1.21–11.21).

## 4. Discussion

The findings of this study reveal a high burden of mental health conditions among children and adolescents undergoing cancer treatment in a resource-limited setting. The prevalence of depression (39.4%) and anxiety (45.3%) aligns with studies from other regions facing unique stressors, such as Israel,^[21,22]^ but is substantially higher than rates reported in high-income countries like the United States and Germany.^[23–25]^ This disparity underscores the profound impact of contextual factors. In our setting, the convergence of limited mental health resources, the inherent stressors of a life-threatening illness, and potential socioeconomic hardships faced by families likely exacerbates psychological distress. The lower prevalence in high-income countries may be attributed to more robust psychosocial support systems integrated into oncology care, highlighting a critical area for intervention in our context.^[25,26]^

In contrast, the prevalence of depression and anxiety in Palestine (Gaza) was reported to be 66.6%,^[27]^ while a study in Israel indicated a prevalence rate of 48%.^[28]^ This variability may stem from the use of different tools to assess depression and anxiety. Additionally, the cutoff values used in these assessments for determining clinically significant symptoms are based on healthy normative populations or pediatric psychiatric populations, which likely differ from those in children with cancer.^[6,29–31]^ The high prevalence rates in Palestine and Israel may also reflect the compounded stress of living in a conflict zone.^[27,28]^ In this study, children and adolescents aged 13 to 18 years were more likely to experience depression compared to those aged 8 to 12 years. This finding aligns with a study conducted in Sweden.^[32]^ One possible explanation is that emotional disorders are more prevalent among adolescents. Depression is estimated to affect 1.1% of adolescents aged 10 to 14 years and 2.8% of those aged 15 to 19 years. Inherited risks, developmental factors, sex hormones, and psychosocial adversity interact, heightening the risk through hormonal influences and disrupted neural pathways.^[11,33]^

The study reveals that children and adolescents who experienced more than 4 weeks of hospitalization are at a greater risk of developing depression compared to those who were hospitalized for <2 weeks. This finding is supported by a study conducted in Palestine (Gaza).^[27]^ Prolonged hospital stays can lead to extended separation from family and friends, which are critical support systems for young patients. This separation often results in feelings of loneliness and a lack of social support, both significant risk factors for depression. Additionally, social and environmental elements play a crucial role; extended stays in medical facilities can create detachment from family and peers, diminishing essential support networks and increasing feelings of isolation.^[34]^ Furthermore, the odds of developing depression were higher among those with an illness duration of more than 24 months compared to those with an illness duration of <6 months. Children and adolescents diagnosed with cancer for longer periods are more likely to experience depression due to disruptions in their social lives and education, persistent physical and mental strain, and chronic stress and anxiety. Missing out on significant developmental stages and social connections often leaves these young patients feeling alone, forlorn, and inadequate.^[35]^

The findings of this study also indicate that corticosteroid medication increases the risk of developing anxiety. This association may be explained by the effects of steroid therapy, which is commonly administered to cancer patients as adjuvant therapy. Such therapy appears to influence emotions and behavior in children with cancer, potentially through the impact of glucocorticoids on the hippocampus.^[36]^ This is further supported by a study conducted in the USA.^[36]^

Additionally, children and adolescents with comorbid illnesses are more likely to develop anxiety than those without comorbidities. This finding aligns with studies conducted in Palestine (Gaza)^[26]^ and England.^[37]^ Children with cancer often experience heightened anxiety, especially when they also have additional medical conditions. This increased anxiety can be attributed to several factors, including the need for additional medical interventions and procedures, which can lead to increased distress. Moreover, children with comorbidities may face unique challenges in their healthcare interactions.^[38]^

Furthermore, the odds of developing anxiety were higher among children and adolescents experiencing severe pain compared to those with no pain. This is consistent with a study conducted in Jakarta, Indonesia, which showed that out of 165 respondents who were children with cancer and experienced hospitalization, 163 (98.7%) reported anxiety due to pain.^[39]^ Pain was identified as the most commonly reported somatic issue among these children, affecting 58%.^[40]^ The possible explanations for this association suggest that cancer manifestations in children and adolescents are frequently accompanied by pain, leading to significant anxiety and distress for both the child and their families. Beyond the impact of the disease itself, young cancer patients may endure considerable pain due to their illness and the medical interventions they receive. These interventions include various procedures such as surgeries, chemotherapy, radiation therapy, and other intrusive measures that can induce discomfort and pain, ultimately resulting in anxiety.^[41–43]^

The high prevalence of anxiety and depression identified in this study calls for the urgent integration of mental health screening and support into standard pediatric oncology care in Ethiopia. In resource-limited settings, task-shifting to trained nonspecialists, such as oncology nurses, can be a feasible strategy for administering brief screening tools like the RCADS.^[44]^ A stepped-care model, aligned with the World Health Organization’s mhGAP guidelines, can be implemented.^[45]^ In this model, all patients are screened at regular intervals. Those with mild symptoms can receive basic psychological first aid and emotional support from trained nurses. Patients scoring above the clinical threshold, like those identified in this study, should be referred to embedded mental health professionals (e.g., psychiatrists or psychologists) for more specialized interventions, such as cognitive-behavioral therapy techniques adapted for the medical setting. Furthermore, training oncologists and pediatricians to recognize the psychological side-effects of treatments like corticosteroids is crucial for proactive management. At a policy level, these findings advocate for the inclusion of mental health support as a core component of national cancer control plans, ensuring budget allocation and sustainable service delivery for this vulnerable population.^[46]^

### 4.1. Limitation

This study has several limitations. The cross-sectional design restricts our ability to establish causal relationships between anxiety, depression, and influencing factors, as we only capture a single point in time. Additionally, we did not consider a comprehensive range of psychosocial stressors, such as family dynamics and socioeconomic status, which may significantly impact mental health outcomes. The study also overlooks potential biological factors, such as hormone levels, which could provide further insights into anxiety and depression in young cancer patients. Moreover, the sample size may limit the generalizability of our findings, particularly if it does not represent the wider population of children with cancer. Lastly, the reliance on self-reported measures may introduce bias, as participants might inaccurately report their symptoms. These limitations underscore the importance of further research utilizing more rigorous designs and broader assessments to better understand the mental health challenges faced by children and adolescents with cancer.

## 5. Conclusion

The prevalence of anxiety and depression among children and adolescents undergoing cancer treatment at Jimma Medical Center is exceptionally high, highlighting the urgent need for attention to these mental health disorders. Key factors contributing to depression include age (13–18 years), prolonged hospitalization exceeding 4 weeks, and illness duration of over 24 months. Additionally, anxiety is notably linked to comorbid conditions, severe pain, and the use of corticosteroids.

Therefore, it is essential to prioritize screening for anxiety and depression in cancer patients to identify those at risk promptly. Implementing integrated management approaches that address both cancer treatment and associated mental health issues at the oncology center will be crucial for improving overall patient care. Furthermore, conducting longitudinal research is necessary to explore the relationships between specific risk factors and the incidence of anxiety and depression, ultimately leading to better-targeted interventions.

## Acknowledgments

We extend our heartfelt gratitude to the nurses working at pediatrics oncology unit, psychiatry unit of JMC for providing and arranging access to the available data, and to the dedicated study teams for their invaluable support during the intervention and data collection process.

## Author contributions

**Conceptualization:** Misikir Tesfaye Dirre, Elias Tesfaye, Alemayehu Negash Torba, Bezaye Alemu Tsegaye, Mubarek Abera Mengistie.

**Data curation:** Misikir Tesfaye Dirre, Elias Tesfaye, Alemayehu Negash Torba, Bezaye Alemu Tsegaye, Tadios Teka Bireda, Tamirat Godebo Woyimo.

**Formal analysis:** Misikir Tesfaye Dirre, Elias Tesfaye, Tamirat Godebo Woyimo.

**Funding acquisition:** Misikir Tesfaye Dirre, Elias Tesfaye.

**Investigation:** Misikir Tesfaye Dirre, Elias Tesfaye, Puturos Puturos Reta.

**Methodology:** Misikir Tesfaye Dirre, Elias Tesfaye, Alemayehu Negash Torba, Bezaye Alemu Tsegaye, Yonas Tesfaye, Mubarek Abera Mengistie, Milki Tufa Feyisa, Puturos Puturos Reta, Tadios Teka Bireda, Tamirat Godebo Woyimo.

**Project administration:** Misikir Tesfaye Dirre, Elias Tesfaye.

**Resources:** Misikir Tesfaye Dirre, Elias Tesfaye, Alemayehu Negash Torba, Bezaye Alemu Tsegaye, Yonas Tesfaye, Mubarek Abera Mengistie, Milki Tufa Feyisa, Puturos Puturos Reta, Tamirat Godebo Woyimo.

**Software:** Misikir Tesfaye Dirre, Elias Tesfaye, Milki Tufa Feyisa, Puturos Puturos Reta, Tadios Teka Bireda, Tamirat Godebo Woyimo.

**Supervision:** Misikir Tesfaye Dirre, Elias Tesfaye, Alemayehu Negash Torba, Bezaye Alemu Tsegaye, Yonas Tesfaye, Mubarek Abera Mengistie.

**Validation:** Misikir Tesfaye Dirre, Elias Tesfaye, Alemayehu Negash Torba, Bezaye Alemu Tsegaye, Yonas Tesfaye, Mubarek Abera Mengistie, Milki Tufa Feyisa, Puturos Puturos Reta.

**Visualization:** Misikir Tesfaye Dirre, Alemayehu Negash Torba, Bezaye Alemu Tsegaye, Yonas Tesfaye, Mubarek Abera Mengistie.

**Writing – original draft:** Misikir Tesfaye Dirre, Alemayehu Negash Torba, Bezaye Alemu Tsegaye, Mubarek Abera Mengistie.

**Writing – review & editing:** Misikir Tesfaye Dirre, Alemayehu Negash Torba, Bezaye Alemu Tsegaye, Yonas Tesfaye, Mubarek Abera Mengistie, Milki Tufa Feyisa, Puturos Puturos Reta, Tadios Teka Bireda, Tamirat Godebo Woyimo.

## References

[1] World Health Organization. Research and development landscape for childhood cancer: a 2023 perspective. World Health Organization, 2023.

[2] Siegel RL Miller KD Jemal A. Cancer statistics, 2018. CA Cancer J Clin. 2018;68:7–30. 29313949 10.3322/caac.21442

[3] Sung H Ferlay J Siegel RL. Global cancer statistics 2020: GLOBOCAN estimates of incidence and mortality worldwide for 36 cancers in 185 countries. CA Cancer J Clin. 2021;71:209–49. 33538338 10.3322/caac.21660

[4] Magrath I Steliarova-Foucher E Epelman S. Paediatric cancer in low-income and middle-income countries. Lancet Oncol. 2013;14:e104–16. 23434340 10.1016/S1470-2045(13)70008-1

[5] Joko‐Fru WY Parkin DM Borok M. Survival from childhood cancers in Eastern Africa: A population‐based registry study. Int J Cancer. 2018;143:2409–15. 29981149 10.1002/ijc.31723

[6] Merikangas KR. Lifetime prevalence of mental disorders in US adolescents: results from the National comorbidity survey replication–adolescent supplement (NCS-A). J Am Acad Child Adolescent Psychiatry. 2010;49:980–9. 10.1016/j.jaac.2010.05.017 PMC2946114 20855043

[7] Widiger TA Crego C. Psychopathy and DSM-5 psychopathology. In: Patrick CJ, ed. Handbook Psychopathy. 2nd ed. Guilford Press; 2018:281–98.

[8] DeJong M Fombonne E. Depression in paediatric cancer: an overview. Psychooncology. 2006;15:553–66. 16355435 10.1002/pon.1002

[9] Evan EE Zeltzer LK. Psychosocial dimensions of cancer in adolescents and young adults. Cancer. 2006;107:1663–71. 16921479 10.1002/cncr.22107

[10] Lee ARYB Low CE Yau CE Li J Ho R Ho CSH. Lifetime burden of psychological symptoms, disorders, and suicide due to cancer in childhood, adolescent, and young adult years: a systematic review and meta-analysis. JAMA Pediatrics. 2023;177:790. 37345504 10.1001/jamapediatrics.2023.2168 PMC10288378

[11] Disease, Communicable. Depression in young people often goes undetected. Practitioner. 2015;259:17–22. 27254891

[12] Ribeiro RC Steliarova-Foucher E Magrath I. Baseline status of paediatric oncology care in ten low-income or mid-income countries receiving My Child Matters support: a descriptive study. Lancet Oncol. 2008;9:721–9. 18672210 10.1016/S1470-2045(08)70194-3 PMC3554242

[13] Klaufus L Verlinden E van der Wal M Kösters M Cuijpers P Chinapaw M. Psychometric evaluation of two short versions of the revised child anxiety and depression Scale. BMC Psychiatry. 2020;20:1–12. 32024481 10.1186/s12888-020-2444-5 PMC7003441

[14] Kösters MP Chinapaw MJ Zwaanswijk M van der Wal MF Koot HM. Structure, reliability, and validity of the revised child anxiety and depression scale (RCADS) in a multi-ethnic urban sample of Dutch children. BMC Psychiatry. 2015;15:132. 26100511 10.1186/s12888-015-0509-7 PMC4477605

[15] Gao LPWC. Influencing factors of quality of life in cancer patients of initial stages receiving radio-and chemotherapy and its countermeasures. Nurs J Chin People’s Lib Army. 2008;8:10–2.

[16] Beuken-Van VD Hj M. Update on prevalence of pain in patients with cancer: systematic review and meta-analysis. J Pain Symptom Manage. 2016;51:1070–90. 27112310 10.1016/j.jpainsymman.2015.12.340

[17] Krebs EE Carey TS Weinberger M. Accuracy of the pain numeric rating scale as a screening test in primary care. J Gen Intern Med. 2007;22:1453–8. 17668269 10.1007/s11606-007-0321-2 PMC2305860

[18] El Rahi C, Zaghloul H, Murillo JR Jr. Pain assessment practices in patients with cancer admitted to the oncology floor. J Hematol Oncol Pharm. 2017;7:109–13.

[19] Dugashvili G. Use of the universal pain assessment tool for evaluating pain associated with temporomandibular disorders in youngsters. Eur J Paediatr Dent. 2019;20:315–9. 31850776 10.23804/ejpd.2019.20.04.11

[20] Bursac Z Gauss CH Williams DK Hosmer DW. Purposeful selection of variables in logistic regression. Source Code Biol Med. 2008;3:1–8. 19087314 10.1186/1751-0473-3-17 PMC2633005

[21] Yardeni M Abebe Campino G Bursztyn S. A three-tier process for screening depression and anxiety among children and adolescents with cancer. Psycho‐Oncol. 2020;29:2019–27. 10.1002/pon.5494 32691478

[22] Lee ARYB Yau CE Low CE Li J Ho RCM Ho CSH. Severity and longitudinal course of depression, anxiety and post-traumatic stress in paediatric and young adult cancer patients: a systematic review and meta-analysis. J Clin Med. 2023;12:1784. 36902569 10.3390/jcm12051784 PMC10003651

[23] Arabiat DH Elliott B Draper P. The prevalence of depression in pediatric oncology patients undergoing chemotherapy treatment in Jordan. J Pediatr Oncol Nurs. 2012;29:283–8. 22907683 10.1177/1043454212451524

[24] Seitz Diana CM Besier T Debatin K-M. Posttraumatic stress, depression and anxiety among adult long-term survivors of cancer in adolescence. Eur J Cancer. 2010;46:1596–606. 20381339 10.1016/j.ejca.2010.03.001

[25] Myers RM Balsamo L Lu X. A prospective study of anxiety, depression, and behavioral changes in the first year after a diagnosis of childhood acute lymphoblastic leukemia: a report from the Children’s Oncology Group. Cancer. 2014;120:1417–25. 24473774 10.1002/cncr.28578 PMC4319360

[26] Anderson ER Mayes LC. Race/ethnicity and internalizing disorders in youth: a review. Clin Psychol Rev. 2010;30:338–48. 20071063 10.1016/j.cpr.2009.12.008

[27] Afifi T. Anxiety and depression among children with cancer, children undergoing hemodialysis and children with thalassemia: a comparative study. Int J Clin Med Educ Res. 2022;1:89–93.

[28] Matziou V Perdikaris P Galanis P Dousis E Tzoumakas K. Evaluating depression in a sample of children and adolescents with cancer in Greece. Int Nurs Rev. 2008;55:314–9. 19522948 10.1111/j.1466-7657.2008.00606.x

[29] Hedström M Ljungman G von Essen L. Perceptions of distress among adolescents recently diagnosed with cancer. J Pediatr Hematol Oncol. 2005;27:15–22. 15654273 10.1097/01.mph.0000151803.72219.ec

[30] Kunin‐Batson AS Lu X Balsamo L. Prevalence and predictors of anxiety and depression after completion of chemotherapy for childhood acute lymphoblastic leukemia: a prospective longitudinal study. Cancer. 2016;122:1608–17. 27028090 10.1002/cncr.29946 PMC4860039

[31] Massie MJ. Prevalence of depression in patients with cancer. JNCI Monographs. 2004;2004:57–71. 10.1093/jncimonographs/lgh014 15263042

[32] Hedström M Haglund K Skolin I von Essen L. Distressing events for children and adolescents with cancer: child, parent, an nurse perceptions. J Pediatr Oncol Nurs. 2003;20:120–32. 12776260 10.1053/jpon.2003.76

[33] World Health Organization. Global accelerated action for the health of adolescents (AA-HA!): Guidance to support country implementation. World Health Organization, 2023.

[34] Mîndru DE. Stress in pediatric patients–the effect of prolonged hospitalization. Med Surg J. 2016;120:417–23. 27483728

[35] Park EM Rosenstein DL. Depression in adolescents and young adults with cancer. Dialogues Clin Neurosci. 2015;17:171–80. 26246791 10.31887/DCNS.2015.17.2/epark PMC4518700

[36] Hochhauser CJ Lewis M Kamen BA Cole PD. Steroid-induced alterations of mood and behavior in children during treatment for acute lymphoblastic leukemia. Support Care Cancer. 2005;13:967–74. 16189647 10.1007/s00520-005-0882-8

[37] Oeffinger KC Mertens AC Sklar CA. Chronic health conditions in adult survivors of childhood cancer. N Engl J Med. 2006;355:1572–82. 17035650 10.1056/NEJMsa060185

[38] Klick JC Ballantine A. Providing care in chronic disease: the ever-changing balance of integrating palliative and restorative medicine. Pediatr Clin North Am. 2007;54:799–812, xii. 17933624 10.1016/j.pcl.2007.07.003

[39] Hasnani F. Analysis of factors causing anxiety in children with cancer experiencing hospitalization. J. Drug Delivery Ther. 2023;13:65–70.

[40] Kaszyński M Lewandowska D Sawicki P Wojcieszak P Pągowska-Klimek I. Efficacy of intravenous lidocaine infusions for pain relief in children undergoing laparoscopic appendectomy: a randomized controlled trial. BMC Anesthesiol. 2021;21:1–11. 33397287 10.1186/s12871-020-01218-0 PMC7784324

[41] Akechi T. Psychiatric disorders in cancer patients: descriptive analysis of 1721 psychiatric referrals at two Japanese cancer center hospitals. Jpn J Clin Oncol. 2001;31:188–94. 11450992 10.1093/jjco/hye039

[42] Bennett M. Pain management for chemotherapy-induced oral mucositis. Nurs Children Young People. 2016;28:25–9. 10.7748/ncyp.2016.e695 27927117

[43] Rata NS Basit M Anggraini S. Hubungan dukungan emosional keluarga dengan tingkat nyeri pada anak acute lymphoblastic leukemia akibat kemoterapi. Jurnal Keperawatan Suaka Insan (JKSI). 2018;3:1–13.

[44] Wiener L Kazak AE Noll RB Patenaude AF Kupst MJ. Standards for the psychosocial care of children with cancer and their families: an evidence-based review. J Clin Oncol. 2015;33:2795–802.

[45] World Health Organization. mhGAP intervention guide for mental, neurological and substance use disorders in non-specialized health settings: version 2.0. World Health Organization; 2016. 27786430

[46] Patel V Saxena S Lund C. The Lancet Commission on global mental health and sustainable development. Lancet. 2018;392:1553–98. 30314863 10.1016/S0140-6736(18)31612-X

